# MrGPS: an m6A-related gene pair signature to predict the prognosis and immunological impact of glioma patients

**DOI:** 10.1093/bib/bbad498

**Published:** 2024-01-03

**Authors:** Ning Zhang, Fengxia Yang, Pengfei Zhao, Nana Jin, Haonan Wu, Tao Liu, Qingshan Geng, Xiaojun Yang, Lixin Cheng

**Affiliations:** Guangdong Provincial Clinical Research Center for Geriatrics, Shenzhen Clinical Research Center for Geriatrics, Shenzhen People’s Hospital, Shenzhen, China; The First Affiliated Hospital of Southern University of Science and Technology, The Second Clinical Medical College of Jinan University; Neuroscience Center, Shantou University Medical College, Shantou, China; Guangdong Provincial Clinical Research Center for Geriatrics, Shenzhen Clinical Research Center for Geriatrics, Shenzhen People’s Hospital, Shenzhen, China; The First Affiliated Hospital of Southern University of Science and Technology, The Second Clinical Medical College of Jinan University; Neuroscience Center, Shantou University Medical College, Shantou, China; Guangdong Provincial Clinical Research Center for Geriatrics, Shenzhen Clinical Research Center for Geriatrics, Shenzhen People’s Hospital, Shenzhen, China; The First Affiliated Hospital of Southern University of Science and Technology, The Second Clinical Medical College of Jinan University; Guangdong Provincial Clinical Research Center for Geriatrics, Shenzhen Clinical Research Center for Geriatrics, Shenzhen People’s Hospital, Shenzhen, China; The First Affiliated Hospital of Southern University of Science and Technology, The Second Clinical Medical College of Jinan University; Guangdong Provincial Clinical Research Center for Geriatrics, Shenzhen Clinical Research Center for Geriatrics, Shenzhen People’s Hospital, Shenzhen, China; The First Affiliated Hospital of Southern University of Science and Technology, The Second Clinical Medical College of Jinan University; International Digital Economy Academy, Shenzhen, China; Guangdong Provincial Clinical Research Center for Geriatrics, Shenzhen Clinical Research Center for Geriatrics, Shenzhen People’s Hospital, Shenzhen, China; The First Affiliated Hospital of Southern University of Science and Technology, The Second Clinical Medical College of Jinan University; Neuroscience Center, Shantou University Medical College, Shantou, China; Guangdong Provincial Clinical Research Center for Geriatrics, Shenzhen Clinical Research Center for Geriatrics, Shenzhen People’s Hospital, Shenzhen, China; The First Affiliated Hospital of Southern University of Science and Technology, The Second Clinical Medical College of Jinan University

**Keywords:** m6A RNA methylation, glioma prognosis, gene expression, iPAGE, immune infiltration

## Abstract

N6-methyladenosine (m6A) RNA methylation is the predominant epigenetic modification for mRNAs that regulates various cancer-related pathways. However, the prognostic significance of m6A modification regulators remains unclear in glioma. By integrating the TCGA lower-grade glioma (LGG) and glioblastoma multiforme (GBM) gene expression data, we demonstrated that both the m6A regulators and m6A-target genes were associated with glioma prognosis and activated various cancer-related pathways. Then, we paired m6A regulators and their target genes as m6A-related gene pairs (MGPs) using the iPAGE algorithm, among which 122 MGPs were significantly reversed in expression between LGG and GBM. Subsequently, we employed LASSO Cox regression analysis to construct an MGP signature (MrGPS) to evaluate glioma prognosis. MrGPS was independently validated in CGGA and GEO glioma cohorts with high accuracy in predicting overall survival. The average area under the receiver operating characteristic curve (AUC) at 1-, 3- and 5-year intervals were 0.752, 0.853 and 0.831, respectively. Combining clinical factors of age and radiotherapy, the AUC of MrGPS was much improved to around 0.90. Furthermore, CIBERSORT and TIDE algorithms revealed that MrGPS is indicative for the immune infiltration level and the response to immune checkpoint inhibitor therapy in glioma patients. In conclusion, our study demonstrated that m6A methylation is a prognostic factor for glioma and the developed prognostic model MrGPS holds potential as a valuable tool for enhancing patient management and facilitating accurate prognosis assessment in cases of glioma.

## INTRODUCTION

Glioma, a prevalent glioneuronal tumor, arises from the malignant proliferation of glial cells and carries a high risk of mortality with a poor prognosis. In the United States, gliomas account for nearly 80% of all central nervous system tumors, resulting in 15 000–17 000 new cases each year [1]. Based on the TCGA classification, gliomas are classified into two categories: lower-grade glioma (LGG) of grade II and III and glioblastoma multiforme (GBM) of grade IV [2, 3]. Patients with LGG have an average overall survival (OS) of about 7 years, whereas those with GBM have a median OS of approximately 1.25 years [4, 5]. Moreover, a majority of patients with LGG will progress to GBM within 5–10 years [6]. Early identification of individuals at high risk of glioma is critical to initiate timely treatment and slow down disease progression. Therefore, it is essential to uncover novel and effective biomarkers for early risk assessment and prognostic prediction.

N6-methyladenosine (m6A) modification had been reported to be closely associated with cancer development, progression, metastasis, recurrence and drug resistance [7, 8]. The development of tumor may result from the dysregulation of the m6A level, which is regulated by three types of regulators, namely, writers (methyltransferases), erasers (demethylases) and readers (m6A-binding proteins). Previous studies have indicated that abnormal m6A modification is involved in glioma initiation and progression [9, 10]. For example, Zhang *et al*. reported an overexpression of the m6A demethylase ALKBH5 in glioblastoma stem-like cells (GSCs). ALKBH5 was observed to demethylate nascent transcripts of the transcription factor FOXM1, consequently leading to an increase in the expression level of FOXM1 [11]. However, the prognostic significance of m6A regulators and m6A-target genes in glioma patients is unclear.

In this study, we performed a systematic analysis of the expression pattern and prognostic relevance of 30 m6A regulators and their target genes in the TCGA database. Specifically, we compared the LGG and GBM samples. Furthermore, we identified the prognostic gene pairs and utilized LASSO Cox regression analysis to build a novel prognostic model named the m6A-related gene pair signature (MrGPS). Time-dependent receiver operating characteristic (ROC) curves and univariate/multivariate Cox regression analysis were used to assess the MrGPS in prognosis in GBM. Finally, we validated MrGPS in five external public cohorts across different platforms.

## MATERIALS AND METHODS

### Data collection

According to previously published literature [10, 12–18] and the RM2 Target database [19], we collected 30 m6A RNA methylation regulators for subsequent analysis, comprising 17 readers, 11 writers and 2 erasers. Detailed information of these regulators is provided in Supplementary Figure S1A available online at http://bib.oxfordjournals.org/.

To train and validate a robust prognostic model, we obtained seven glioma gene expression datasets along with their respective clinical information from three sources, the UCSC Xena database (http://xena.ucsc.edu/) [20], Gene Expression Omnibus (GEO, https://www.ncbi.nlm.nih.gov/geo) [21] and the Chinese Glioma Genome Atlas (CGGA, http://www.cgga.org.cn) [22].

TCGA glioma cohorts were integrated and analyzed as the discovery set, including 529 LGG samples and 168 GBM samples. These samples were randomly divided into two datasets: the training set comprising 488 samples (70%) and the test set comprising 209 samples (30%). Five external cohorts, namely, CGGA-325 (*N* = 325), CGGA-693 (*N* = 693), CGGA-301 (*N* = 301), GSE4412 (*N* = 85) and GSE43378 (*N* = 50), were used as validation purposes (Table 1).

**Table 1:** Discovery and validation datasets of RNA expression

Cohorts	Platforms	Technique description	Sample size	LGG	GBM
Discovery					
TCGA	Illumina HiSeq 2000	RNA seq	697	529	168
Validation					
CGGA-693	Illumina HiSeq 4000	RNA seq	693	443	249
CGGA-325	Illumina HiSeq 2000/2500	RNA seq	325	182	139
CGGA-301	Agilent-014850 Whole Human Genome Microarray (GPL4133)	Microarray	301	174	124
GSE43378	Affy HG-U133 Plus 2.0 (GPL570)	Microarray	50	18	32
GSE4412	Affy HG-U133 Plus 2.0 (GPL96)	Microarray	85	26	59

To investigate the expression pattern of m6A-related gene pairs (MGPs) in normal brain, we also downloaded the expression data for normal brain tissue from Genotype Tissue Expression (GTEx, https://gtexportal.org/home/).

### The t-distributed stochastic neighbor embedding analysis

The t-distributed stochastic neighbor embedding (t-SNE) is a non-parametric, unsupervised method used to separate patients into distinct clusters. In order to investigate the prognostic significance of m6A regulators, we employed the R/Bioconductor package ‘Rtsne’ to perform t-SNE analysis on the total glioma samples and assess whether the expression of these regulators could differentiate LGG and GBM patients.

### Identification of m6A regulators with prognostic value

In the TCGA LGG and GBM datasets, we calculated the hazard ratios (HRs) of m6A regulators in glioma patients using univariate Cox proportional hazard regression. Regulators with *P* < 0.05 and HR > 1 were considered as a risk factor; regulators with *P* < 0.05 and HR < 1 were considered as a protective factor.

### Identification of m6A-target genes

To retrieve the m6A-target genes, we divide expression profile into two parts: one comprising the m6A regulator expression matrix and the other consisting of the gene expression matrix of non-regulators. Then, we calculated the Pearson correlation coefficient (PCC) between every regulator and non-regulator from the two matrices as follows:


(1) \begin{equation*} \mathrm{PCC}=\frac{\sum_{i=1}^n\left({M}_i-\overline{M}\right)\left({G}_i-\overline{G}\right)}{\sqrt{\sum_{i=1}^n{\left({M}_i-\overline{M}\right)}^2}\sqrt{\sum_{i=1}^n{\left({G}_i-\overline{G}\right)}^2}} \end{equation*}

where ${M}_i$ represents the expression level of each m6A regulator and ${G}_i$ represents the expression level of genes other than m6A regulators (non-regulators). A gene is defined as an m6A target gene if it significantly correlates with at least one regulator (|PCC| > 0.5 and *P*-value <0.01).

### Gene set enrichment analysis

We utilized the R/Bioconductor package ‘limma’ to calculate the fold change of m6A-target genes. Then, we ranked these genes based on the fold change and conducted gene set enrichment analysis (GSEA) using the R/Bioconductor package ‘clusterProfiler’, to investigate the KEGG signaling pathways regulated by these genes [23]. The GSEA results were represented by positive and negative normalized enrichment score (NES), respectively, indicating higher and lower expression in different gene sets tested. A significance cutoff for pathway enrichment was established as |NES| > 1 and adjusted *P*-value <0.05, ensuring robustness of the findings.

### Construction of MGPs

To circumvent the limitation of the unreliable absolute expression value and the possible batch effect introduced by integrating multiple cohorts [24], we applied the individualized Pair Analysis of Gene Expression (iPAGE) [25–30], which uses the relative expressions of gene pairs, to establish a prognostic model. The first step in implementing iPAGE is to build gene pairs. We combined each m6A regulator (*M*) with its corresponding target gene (*G*) to form an MGP, which may work together in regulating gene expression to further influence cancer progression. The relative expression value of each MGP in each patient was defined as follows:


(2) \begin{equation*} I\left(\mathrm{MGP}\right)=\left\{\begin{array}{@{}l}1,\mathrm{if}\ E(M)\ge E(G)\\{}-1,\mathrm{if}\ E(M)<E(G)\end{array}\right.\!\!. \end{equation*}

where *E*(*X*) represents the expression intensity of gene *X*. If *E*(*M*) ≥ *E*(*G*), the relative expression value is 1; otherwise, the value is −1.

${R}^i$ is defined as a vector constituted by the value of MGPs in the LGG group,


(3) \begin{equation*} {R}^i=\left(I\left({\mathrm{MGP}}_1\right),I\left({\mathrm{MGP}}_2\right),\dots, I\left({\mathrm{MGP}}_m\right)\right);i\in \left\{1,m\right\}. \end{equation*}

${R}^j$ is a vector constituted by the value of MGPs in the GBM group,


(4) \begin{equation*} {R}^j=\left(I\left({\mathrm{MGP}}_{m+1}\right),I\left({\mathrm{MGP}}_{m+2}\right),\dots, I\left({\mathrm{MGP}}_n\right)\right);j\in \left\{m+1,n\right\}. \end{equation*}

For each MGP, the number of LGG samples with *E*(*M*) ≥ *E*(*G*) is calculated as follows:


(5) \begin{equation*} a=\frac{1}{2}{\sum}_{i=1}^m\left({R}^i+1\right), \end{equation*}

and the number of LGG samples with *E*(*M*) < *E*(*G*) is


(6) \begin{equation*} b=\frac{1}{2}{\sum}_{i=1}^m\left(1-{R}^i\right), \end{equation*}

the number of GBM samples with *E*(*M*) ≥ *E*(*G*) is


(7) \begin{equation*} c=\frac{1}{2}{\sum}_{j=m+1}^n\left({R}^j+1\right), \end{equation*}

the number of GBM samples with *E*(*M*) < *E*(*G*) is


(8) \begin{equation*} d=\frac{1}{2}{\sum}_{j=m+1}^n\left(1-{R}^j\right), \end{equation*}

where $m$ indicates the number of LGG patients and $n-m$ represents the number of GBM patients.

Afterward, Fisher’s exact test was performed for each MGP, where the *P*-value was calculated by


$$ p=\frac{\left(\begin{array}{c}a+b\\{}a\end{array}\right)\left(\begin{array}{c}c+d\\{}c\end{array}\right)}{\left(\begin{array}{c}n\\{}a+c\end{array}\right)} $$

(9) \begin{equation*} =\frac{\left(a+b\right)!\left(c+d\right)!\left(a+c\right)!\left(b+d\right)!}{a!b!c!d!n!}, \end{equation*}

where $n$ is the number of glioma patients, including both LGG and GBM.

MGPs with *P* < 1e−70 were selected as candidate gene pairs to predict OS of glioma patients.

### Construction of MrGPS

**Figure 1:**
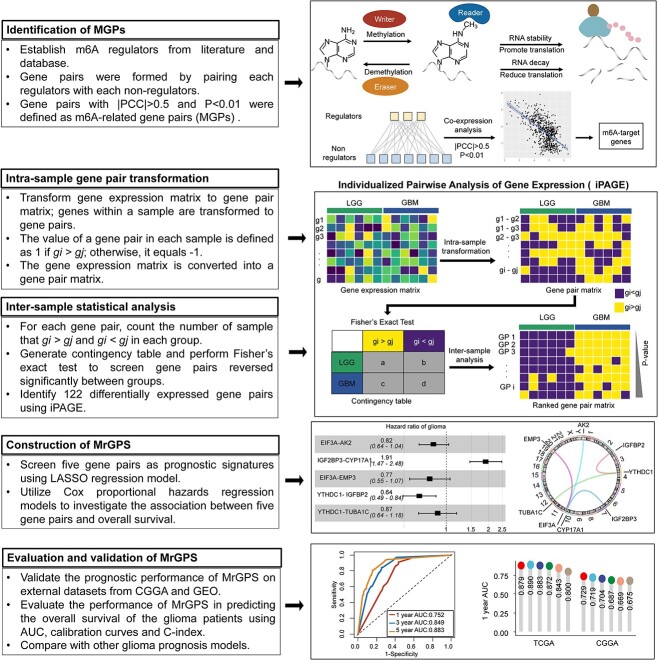
Workflow of this study. First, we established m6A regulators from literature and the database. A gene pair was formed by combining a regulator and a non-regulator. Gene pairs with |PCC| > 0.5 and *P* < 0.01 were defined as MGPs. Then, based on iPAGE algorithm, MGPs were identified as dysregulation signatures in the glioma development and the relative expression of each pair was calculated. Next, LASSO Cox regression analyses were conducted to screen the most significant prognostic gene pairs and construct a gene pair prognostic model. Finally, we assessed the prognostic capabilities in five independent cohorts and compared model performance with other five prognostic models.

We utilized the R package ‘glmnet’ [31] to perform LASSO Cox analysis and screen the candidate prognostic MGPs. We used the selected prognostic gene pairs to construct a multivariate Cox analysis that enabled us to establish a prediction model for prognosis. Detailed methods and procedures are shown in Figure 1. Finally, the risk score was calculated as follows:


(10) \begin{equation*}\mathrm{Risk}\ \mathrm{score}=\sum \left({\mathrm{coef}}_i\ast I\left({\mathrm{MGP}}_i\right)\right) \end{equation*}

### Performance validation

We assessed the prognostic value of the MrGPS in both the independent GEO datasets and CGGA datasets. Kaplan–Meier (KM) analysis was conducted to estimate the OS of the low-risk and high-risk cohorts using the R packages ‘survminer’ and ‘survival’. To further investigate the specificity and sensitivity of MrGPS for 1-, 3- and 5-year survival prediction, we adopted the ‘timeROC’ R package to conduct time-dependent ROC curves. The predictive power of the risk models was estimated by calculating the area under the ROC (AUC). We plotted the calibration curve of the MrGPS using an R package ‘rms’ to visualize agreement between predicted and observed survival probabilities. We determine the discrimination of our model by using R package ‘survival’ to calculate the concordance index (C-index).

### Immune landscape analysis

To assess the differences in the tumor microenvironment (TME) between the high- and low-risk groups, we utilized the Cell-type Identification by Estimating Relative Subsets of RNA Transcripts (CIBERSORT) algorithm [32] to estimate the proportions of 22 types of immune cells. In addition, we calculated the stroma score, immune score, estimate score and tumor purity of each sample using ‘estimate’ R package [33, 34].

Accumulated evidence showed that the dysregulation of five immune checkpoints, CTLA4, CD274, PDCD1, LAG3 and HAVCR2, leads to immune exhaustion [35]. Thus, we explored the expression level and their association with OS of the five immune checkpoints within the two risk groups [36, 37]. We employed the tumor immune dysfunction and exclusion (TIDE) algorithm (http://tide.dfci.harvard.edu/) [38, 39] to evaluate T-cell dysfunction and exclusion in patients. This assessment was based on three cell types known to restrict tumor T-cell infiltration: the M2 subtype of tumor-associated fibroblasts (CAFs), myeloid-derived suppressor cells (MDSCs) and the M2 subtype of tumor-associated macrophages (TAM.M2). The TIDE scores, obtained from the TIDE algorithm, is a reliable indicator of tumor immune escape and resistance to cancer immunotherapies. The patients with higher TIDE score are more likely to become resistance to cancer immunotherapies.

### Statistical analysis

All statistical analyses were conducted using R software, version 4.2.1 (http://www.r-project.org/). The expression levels of m6A RNA methylation regulators between LGG and GBM patients were compared using Student’s *t*-test. To assess the correlation between m6A RNA methylation regulators and other genes, PCCs were employed. Fisher’s exact test was applied to determine the significantly reversed MGPs between the LGG and GBM groups. To determine the prognostic determinants from m6A regulators and MGPs, univariate and multivariate Cox analyses were performed. KM survival analysis and the log-rank test were used to evaluate the OS of glioma patients. The performance of the model was assessed using AUC, calibration curve and C-index, which were calculated using the ‘timeROC’, ‘survcomp’ and ‘rms’ packages.

## RESULTS

### Transcriptomic landscape and functional characterization of m6A regulators in glioma

**Figure 2:**
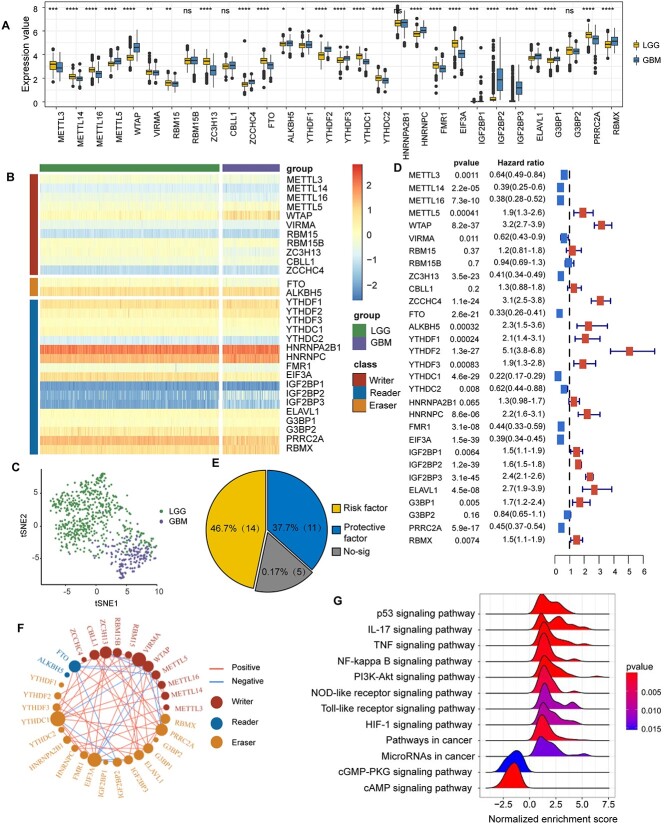
Characterization of the m6A regulators in glioma prognosis. (**A**) Expression difference of the 30 m6A regulators between LGG and GBM. (**B**) Heatmap showing the expression pattern of the writers, erasers and readers. (**C**) t-SNE analysis illustrating the LGG and GBM patients. (**D**) Prognostic value of the m6A regulators. (**E**) Proportion of protective factors and risk factors of the m6A regulators. (**F**) Connection of the m6A regulators in glioma. Edge represents the expression correlation between regulators, and vertex size represents the degree of each regulator. Edges with absolute PCC larger than 0.5 are shown in the network. (**G**) Representative enriched KEGG pathways for the m6A-target genes. The peak value represents normalized enrichment score (NES); NES > 1 represents that the pathway is upregulated while NES < 1 represents that it is suppressed. GSEA curves showing the KEGG pathways enriched by m6A-target genes.

Previous studies have indicated the involvement of various m6A reading proteins in the development of glioma [40]. Therefore, we integrated transcriptome data of LGG and GBM patients sourced from the TCGA dataset to explore the differential expression of m6A regulators. A majority of these regulators showed significantly differential expression between LGG and GBM patients. Notably, IGF2BP1, IGF2BP2 and IGF2BP3, belonging to the insulin-like growth factor-2 mRNA-binding protein class [41], displayed extreme overexpression (fold change >8) in GBM patients (Figure 2A and B). Our finding aligns with previous experimental studies, demonstrating a general overexpression of IGF2BPs across various cancer types [42]. The results highlight the potential oncogenic role of IGF2BPs in glioma. As expected, LGG and GBM patients were distinctly categorized into two groups based on the expression of m6A regulators, as revealed by t-SNE analysis (Figure 2C).

Next, we evaluated the association between the m6A regulators and glioma prognosis using univariate Cox regression. Totally, 25 (83.3%) regulators were identified as significantly factors associated with a glioma prognosis. Among them, 14 regulators were detected as risk factors, including three writers (METTL5, WTAP and ZCCHC4), 10 readers (YTHDF1, YTHDF2, YTHDF3, HNRNPC, IGF2BP1, IGF2BP2, IGF2BP3, ELAVL1, G3BP1 and RBMX) and one eraser (ALKBH5), while 11 regulators were identified as protective factors, including five writers (METTL3, METTL14, METTL16, VIRMA and ZC3H13), five reads (YTHDC1, YTHDC2, FMR1, EIF3A and PRRC2A) and one eraser (FTO) (Figure 2D and E). Then, we examined the expression correlation among these m6A regulators to explore their co-expression pattern (Figure 2F). Interestingly, positive correlations were observed in most of the regulators, except for two writers, WTAP and METTL5, which showed negative correlation with EIF3A, FTO, PRRC2A and ZC3H13.

m6A methylation may regulate RNA stability and decay [43], suggesting that the expression of the m6A regulator tends to be correlated with the expression of target genes. Therefore, we hypothesize that genes showing significant correlation with m6A regulators have the potential to act as candidate m6A-target genes. We calculated the PCC between the expression level of the 30 m6A regulators and other 12 298 genes (non-regulators) commonly detected across platforms and obtained 368 940 gene pairs. Then, we identified 19 374 MGPs with |PCC| > 0.5 and *P* < 0.01. Among them, we determined 5731 unique m6A-target genes and performed differential expression analysis. Utilizing GSEA, we discovered that the up-regulated target genes were primarily associated with classical carcinogenesis pathways and immune response pathways, such as the p53, IL 17, TNF, NF-kappa B, PI3K-Akt, NOD-like receptor and TOLL-like receptor signaling pathways, suggesting that m6A modification increases glioma proliferation and immune response (Figure 2G). For the down-regulated target genes, they are primarily implemented in cGMP-PKG and cAMP signaling pathways. The lower level of cAMP/cGMP may result from some tumor cells overexpressing phosphodiesterases [44].

### Construction of prognostic model using iPAGE

Among all 368 940 gene pairs identified in the last section, approximately one-third (115 594) were verified in the RM2 database. For the 19 374 MGPs identified from the previous section, 6919 MGPs were verified by CLIP-seq. Using the hypergeometric test, we found the number of verified MGPs is significantly higher than expected (*P* < 5.7e−41) (Supplementary Figure S1B available online at http://bib.oxfordjournals.org/).

**Figure 3:**
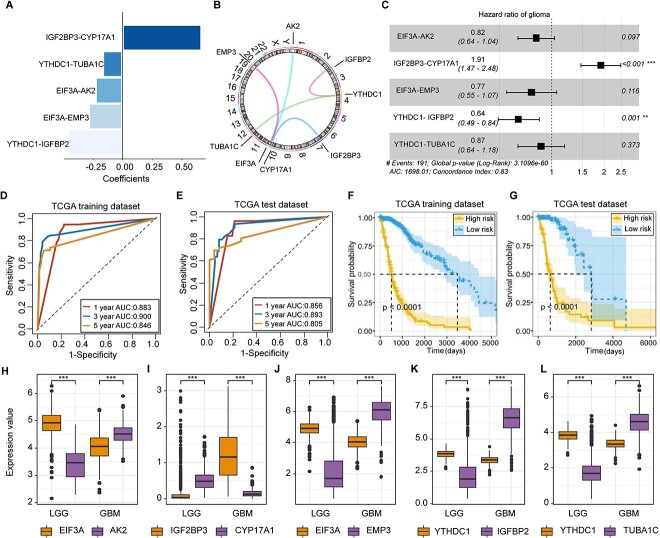
Construction of MrGPS. (**A**) The coefficient of five gene pairs in the model. (**B**) Circular diagram showing the location of eight genes on 23 chromosomes. (**C**) OS HRs of five gene pairs. (**D**, **E**) Time-dependent ROC curves showing the AUC values of 1-, 3- and 5-year OS in the TCGA training set and test set. (**F**, **G**) Comparison of the OS between high- and low-risk groups in the TCGA training set and test set. (**H**–**L**) Comparison of the relative expression value of five gene pairs between LGG and GBM. The expression relationship of each gene pair was reversed between LGG and GBM.

Using iPAGE, we directly combined the samples among the GDC TCGA GBM cohort and GDC TCGA LGG cohort, rather than normalized the expression value of samples from different cohort, which is an advantage of iPAGE in data integration skipping the frustrating steps of data preprocessing [45–47]. Ultimately, 122 MGPs with significant phenotype association (GBM versus LGG) were filtered for further analysis (*P* < 1e−70, Fisher’s exact test) (Supplementary Figure S1C available online at http://bib.oxfordjournals.org/). After that, we calculated the proportion of each MGPs in which the expression level of regulator is higher than target gene in normal brain, LGG and GBM samples, respectively. The result showed a gradually decreasing trend of the proportion in the normal, LGG and GBM samples, suggesting that these MGPs were highly associated with the progression of glioma (Supplementary Figure S1D available online at http://bib.oxfordjournals.org/). Subsequently, we employed LASSO Cox analysis to prioritize the MGPs that are linked to the prognosis of glioma. Through multivariate Cox ratio hazard regression analysis, we identified five MGPs that constituted the prognostic model in the training set. We defined the model as MrGPS as follows (Figure 3A):


\begin{eqnarray*} &&\kern-1.5pc\mathrm{Risk}\ \mathrm{score}=-{0.204}{\times}\ \left(\mathrm{EIF}3\mathrm{A}-\mathrm{AK}2\right)-{0.266}{\times}\ \left(\mathrm{EIF}3\mathrm{A}-\mathrm{EMP}3\right)\\&&-{0.439}{\times}\ \left(\mathrm{YTHDC}1-\mathrm{IGFBP}2\right)-{0.140}{\times}\ \left(\mathrm{YTHDC}1-\mathrm{TUBA}1\mathrm{C}\right)\\&&+{0.647}{\times}\ \left(\mathrm{IGF}2\mathrm{BP}3-\mathrm{CYP}17\mathrm{A}1\right). \end{eqnarray*}

The chromosomal distribution of the five gene pairs was depicted in the circos plot (Figure 3B). The HR of each gene pair in MrGPS was summarized in a forest plot (Figure 3C). The global *P*-value of MrGPS showed a significance in the association between MrGPS and the prognosis of glioma. In the training dataset, the AUC values for predicting 1-, 3- and 5-year survival time were 0.883, 0.900 and 0.846, respectively; in the test dataset, the corresponding AUCs were 0.856, 0.893 and 0.805, respectively (Figure 3D and E). Our model indicated that glioma patients with a higher risk score had a poorer prognosis for glioma compared to patients in the low-risk group (*P* < 0.0001, log-rank test) (Figure 3F and G).

We also compared the relative changes of gene expression level of each gene pair between LGG and GBM patients (Figure 3H–L). We noted that, in four gene pairs (EIF3A-AK2, EIF3A-EMP3, YTHDC1-IGFBP2, YTHDC1-TUBA1C), the expression level of the m6A regulator is significantly higher than its target gene in the LGG patients, whereas the expression of IGF2BP3 is up-regulated in GBM patients. These data showed that IGF2BP3 might serve as a potential cancerogenic regulator to promote glioma progression.

### External validation of MrGPS

**Figure 4:**
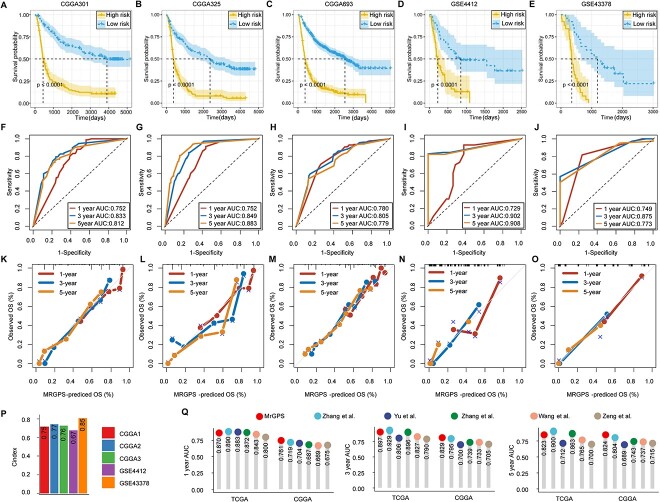
Validation of MrGPS in different type of datasets. (**A**–**E**) KM curves of two risk groups in five validation datasets. (**F**–**J**) Time-dependent ROC curves for 1-, 3- and 5-year OS in validation cohorts. (**K**–**O**) Calibration curves for testing the agreement between 1-, 3- and 5-year predicted OS and actual observations. (**P**) C-index in five validation cohorts. (**Q**) Performance comparison of MrGPS and other signatures in TCGA and CGGA datasets. MrGPS performed better than all the other model in the CGGA dataset.

We assessed the performance and robustness of our MrGPS prognostic model in three CGGA datasets and two GEO datasets, detected by five different platforms (Table 1). The survival probability of patients predicted using the risk score in the five validation cohorts exhibited a significant difference between high-risk and low-risk groups, stratified based on the median risk score. Patients with a high-risk score exhibited a poorer OS compared to those with a low-risk score (*P* < 0.01, Figure 4A–E).

We then used time-dependent ROC analysis and AUC to evaluate the performance of the model at different follow-up times. In the CGGA-301 dataset, the AUC values of 1-, 3- and 5-year OS were 0.752, 0.833 and 0.812, respectively. In the CGGA-325 dataset, the AUC values of the three time points were 0.752, 0.849 and 0.883, respectively. In the CGGA-693 dataset, the corresponding AUC values were 0.780, 0.805 and 0.779. In the GSE4412 dataset, the AUC values were 0.729, 0.902 and 0.908. In the GSE43378 dataset, the AUC values were 0.749, 0.875 and 0.773 (Figure 4F–J). In particular, MrGPS maintained a consistently high performance across all validation datasets, regardless of the transcriptomic quantification techniques utilized, including RNA-sequencing or microarray. The calibration curves exhibited a highly satisfactory degree of congruity between the predicted OS probabilities predicted and those that were observed in the respective validation datasets (Figure 4K–O). In addition, the C-indexes of our MrGPS model for predicting OS were 0.75, 0.77, 0.76, 0.67 and 0.85 in the five external datasets (Figure 4P). Finally, we compared the average AUC of MrGPS with other prognostic models in TCGA and CGGA datasets. In the CGGA validation datasets, MrGPS showed a higher performance than all other models, whereas a comparable performance was observed across models in TCGA datasets [48–53] (Figure 4Q).

Besides, we constructed a 20-gene prognostic signature via classical LASSO Cox regression using individual genes, instead of curated gene pairs. This signature was demonstrated to be inferior to MrGPS in all the five validation datasets (Supplementary Figure S2A–G available online at http://bib.oxfordjournals.org/). The results further confirmed that prognostic model adapted the iPAGE algorithm is superior to traditional methods and MrGPS may serve as a consistent prognostic indicator across diverse datasets and platforms.

### MrGPS is associated with immune infiltration

**Figure 5:**
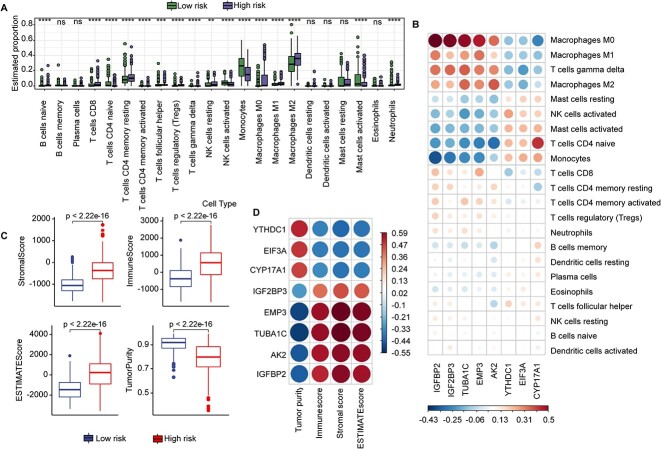
Immune microenvironment characteristics in low- and high- risk groups. (**A**) Abundance of 22 immune cells estimated by CIBERSORT algorithm in two groups. (**B**) Correlation matrix of the eight genes and 22 immune cells. (**C**) Stroma score, immune score, ESTIMATE score and tumor purity in high- and low-risk groups. (**D**) Correlation matrix of eight genes and stroma score, immune score, ESTIMATE score and tumor purity. ^*^P < 0.05; ^*^^*^P < 0.01; ^*^^*^^*^P < 0.001; ^*^^*^^*^^*^P < 0.0001; ns, not significant.

Immune infiltration plays complex and multifaceted roles in glioma, holding great significance in the field of oncology. We hypothesize that MrGPS, as a robust prognostic indicator for glioma, should maintain the association with immune infiltration. Thus, we calculated and compared the fractions of 22 immune cell types using the CIBERSORT algorithm. The results indicated that CD4 T cells, monocytes, macrophages and mast cells constituted the main infiltrating immune cells. In comparison to the low-risk group, the high-risk group exhibited a significantly higher proportion of resting CD4 memory T cells and M2 macrophages. Conversely, the low-risk group showed a notable infiltration of monocytes and activated mast cells (*P* < 0.01; Figure 5A). We evaluated the association between MrGPS and immune infiltration by assessing the correlation between eight MrGPS genes and 22 immune cell types. We observed that CYP17A1 exhibited a negative correlation with M0 macrophages but a positive correlation with CD4 naive T cells (Figure 5B). Similarly, IGFBP2, IGF2BP3, TUBA1C, EMP3 and AK2 demonstrated a positive association with M0 macrophages while displaying a negative correlation with monocytes and CD4 naive T cells.

Next, we evaluated the immune infiltration using four scores, namely, stroma score, immune score, estimate score and tumor purity. The high-risk group exhibited significantly elevated stroma, immune and estimate scores, whereas the low-risk group showed increased tumor purity. This implies that the high-risk group had a greater degree of immune infiltration compared to the low-risk group (Figure 5C). Overall, an inverse correlation was noted between tumor purity and the expression levels of IGF2BP3, EMP3, TUBA1C, AK2 and IGFBP2 (average PCC = −0.48). Conversely, a positive association was found between tumor purity and the expression levels of YTHDC1, EIF3A and CYP17A1 (average PCC = 0.42) (Figure 5D). In summary, these results suggest that MrGPS play potential roles in immune infiltration, which may further influence the glioma prognosis.

### Evaluation of ICI treatment response of MrGPS

**Figure 6:**
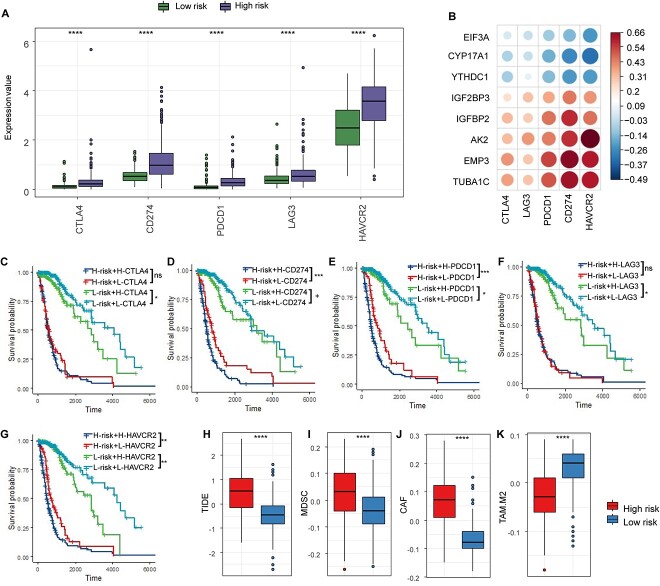
Association between MrGPS and immunotherapy. (**A**) Comparison of the expression of five immune checkpoints in high- and low-risk groups. (**B**) Correlation matrix of eight genes and five immune checkpoints. (**C**–**G**) KM survival curve shows OS among four patient groups stratified by the MrGPS and immune checkpoints. (**H**–**K**) TIDE analysis results in high- and low-risk groups. +*P* < 0.1, ^*^P < 0.05, ^*^^*^P < 0.01, ^*^^*^^*^P < 0.001 and ^*^^*^^*^^*^P < 0.0001. ns, not statistically significant.

As immunotherapy shows promise as a cancer treatment, we conducted an investigation into the connection between immune checkpoints and risk stratification in glioma. Our analysis revealed that the expression of the five immune checkpoints, namely, CTLA4, CD274, PDCD11, LAG3 and HAVCR2, were significantly overexpressed in the high-risk group compared with the low-risk group (*P* < 0.01, Figure 6A). Negative correlations were detected between immune checkpoints and three MrGPS model genes (EIF3A, CYP17A1 and YTHDC1), while a positive correlation was detected between immune checkpoints and the other five genes (IGF2BP3, IGFBP2, AK2, EMP3 and TUBA1C) (Figure 6B).

In the low-risk group, patients with lower expression level of immune checkpoints had a significantly better prognosis than those with high expression of immune checkpoints, indicating that the high expression level of immune checkpoints is a risk factor for glioma prognosis. Similar prognostic difference was observed in the high-risk group, although expression of CTLA4 and LAG3 showed no significance in classifying the patients (Figure 6C–G). To validate the aforementioned findings, we utilized the TIDE algorithm to predict immunotherapy response in both low- and high-risk patient groups. The outcomes demonstrated a significantly elevated TIDE score in the high-risk group compared to the low-risk group. The two distinct risk groups exhibited varying levels of three T-cell-exclusion signatures. Specifically, the high-risk group displayed a higher abundance of MDSC and CAF (*P* < 0.001), while TAM.M2 was observed at lower levels in the high-risk group. These results reveal that the low-risk group had a low TIDE score, which is more likely to benefit from ICI therapy and improved survival after immunotherapy. (Figure 6H–K).

### Combining the MrGPS with clinical factors

**Figure 7:**
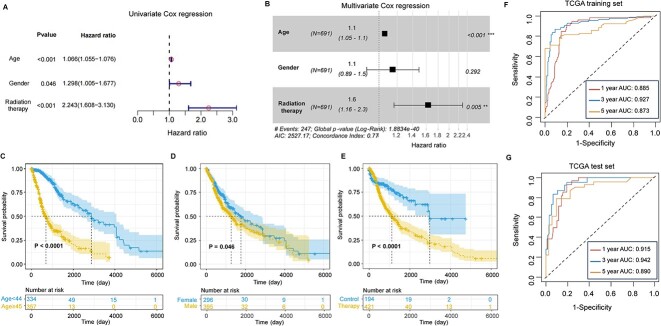
Prognostic value of clinical characteristics. (**A**) Univariate Cox regression for survival analysis according to clinical characteristics. (**B**) KM curves for patients younger than age 45 and patients over 45 years. (**C**) KM curves for male patients and female patients. (**D**) KM curves for patients received radio therapy and not received. (**E**) Multivariate Cox regression for survival analysis according to clinical characteristics. AUC curves showing the performance of MrGPS combining clinical features in the TCGA training set (**F**) and test set (**G**).

In addition to molecular features, we considered available clinical factors to enhance the predictive performance of MrGPS. We found that age, gender and radiotherapy were independent prognostic factors via univariate analysis in the TCGA dataset (Figure 7A). The age stratified the glioma patients older or less than 45 into significantly different prognostic groups. Patients older than 45 have significantly worse survival outcome than the youth ones (*P* < 0.0001, LogRank test). Our results also demonstrated that male patients (*P* < 0.05, LogRank test) and patients with radiotherapy (*P* < 0.0001, LogRank test) have a much poor prognosis (Figure 7B–D).

In our multivariate Cox regression analysis, we discovered significant associations between glioma prognosis and age as well as radiotherapy, alongside gender (*P* < 0.001 and *P* < 0.005, LogRank test, Figure 7E). To further improve model accuracy, we combined age, radiotherapy and the five gene pairs in MrGPS to fit a Cox proportional hazards regression model using the TCGA dataset and derived a new prognostic index for glioma (Figure 7F and G). In the TCGA test set, the AUCs of 1-, 3- and 5-year OS prediction using the new prognostic index achieved 0.915, 0.942 and 0.890, respectively, which are much increased than that of MrGPS, indicating the importance of clinical factors in glioma prognosis estimation.

## DISCUSSION

Extensive evidence supports the association of aberrant m6A modification with tumorigenesis, tumor progression and the anti-tumor immune response [54]. Various m6A regulators modulate the decay or stability of downstream target genes, affecting cancer development and progression. For example, YTHDF2 promotes glioma malignancy by degrading UBXN1 mRNA, while METTL3-mediated m6A modification enhances glioma metastasis by stabilizing PCBP2 [55, 56]. Previous studies have reported that certain m6A regulators serve as important prognostic markers in various types of cancers. For instance, WTAP is overexpressed in hepatocellular carcinoma and drives liver cancer development [57]. Low expression of METTL14 has been linked to an unfavorable prognosis among colorectal cancer patients. Furthermore, experiments involving the knockdown of METTL14 have revealed a significant enhancement in the proliferative and invasive capabilities of colorectal cancer cells [58]. However, only a few studies have reported the prognostic value of m6A regulators in glioma [59]. Therefore, it is crucial to develop a novel m6A-based signature to elucidate the molecular mechanisms of glioma and improve its prognosis.

The iPAGE strategy is a powerful tool for identifying gene pair signatures for diagnosis and prognosis of various diseases [25–30, 60]. The reverse gene pairs constructed by the iPAGE method enhances the robustness of the prognostic model and reduces the impact of batch effects from different laboratories and platforms [25, 28]. In this study, we observed dysregulation of multiple m6A methylation regulators in both LGG and GBM patients, suggesting the involvement of m6A regulation disorder in the development and progression of glioma. We also explored m6A-target genes using correlation analysis and GSEA analysis and found that they were enriched in cancer-related pathways. We then established MGPs using the iPAGE method and subsequently developed an MrGPS to predict the OS of glioma patients. MrGPS showed high performance in glioma prognosis with an average C-index of 0.76 and an average AUC of 0.82 in the 1-, 3- and 5-year OS predictions in five independent cohorts, which outperformed the state-of-the-art models [49–53].

Eight genes in the model showed significant differences between LGG and GBM, including three regulators (EIF3A, IGF2BP3, YTHDC1) and five target genes (AK2, CYP17A1, EMP3, IGFBP2 and TUBA1C). Among these gene pairs, interestingly, only IGF2BP3-CYP17A1 was identified as a risk factor, with an HR above 1, making it a key gene pair in our model.

In MrGPS, the three m6A regulators are all readers and previous studies have described that EIF3A is overexpressed in malignant tumor cells and promote tumor cell proliferation [61]. Similarly, IGF2BP3 has been reported to promote glioma progression and migration [62]. YTHDC1 affects glioma cell proliferation, cell cycle and apoptosis via the JAK-STAT pathway [63].

Among the target genes in MrGPS, CYP17A1 is up-regulated in gliomas, playing a role in mediating glioma cell invasiveness and contributing to resistance against TMZ-induced cytotoxicity [64]. EMP3 regulates membrane receptors associated with IDH-wild type glioblastoma [65, 66]. On the other hand, IGFBP2 activation stimulates the PTEN, AKT and related pathways, ultimately leading to increased invasiveness and malignancy [67–69]. Furthermore, TUBA1C demonstrates up-regulation in glioma tissues in comparison to normal brain tissues. Notably, knockdown of TUBA1C suppressed proliferation and migration, along with the induction of apoptosis and G2/M phase arrest in glioma cells [70].

We also compared the tumor immune microenvironment and immune response between high- and low-risk groups and found that the risk score is an indicator of the immune infiltration level and ICI therapy response. IGF2BP3, IGFBP2, AK2, EMP3 and TUBA1C in MrGPS were positively associated with five immune checkpoint genes. Specifically, IGF2BP3 was reported to be associated with PD-L1 and promote stabilization of PD-L1 mRNA by METTL3-mediated m6A modification in breast cancer [71]. IGFBP2 has been shown to promote PD-L1 expression by activating the EGFR-STAT3 signaling pathway in malignant melanoma [72]. These results suggest that IGF2BP3 and IGFBP2 may have important implications in glioma immunotherapy.

In conclusion, we developed a five-gene pair signature using the iPAGE method to predict the prognosis of glioma patients and assist clinical management. Our approach, which utilizes the relative expression of gene pairs, offers a significant advancement over models based on individual genes, enabling data integration and batch-effect mitigation [73–75]. MrGPS is associated with immune infiltration, immune checkpoints and response of ICI therapy, suggesting that these factors may play crucial roles in influencing the prognosis of glioma. Although good performance was achieved, these results need to be confirmed by further cell experiments and clinical studies to elucidate the biological role of these gene pairs in future work.

**Key Points**

- m6A methylation is associated with glioma prognosis at the transcriptome level.
- m6A-related gene pairs identified by the iPAGE algorithm are effective in predicting the overall survival for glioma patients across platforms and cohorts.
- MrGPS is a potential prognosis predictor for glioma, and it correlates with tumor immune microenvironment and immunotherapy response.

## Supplementary Material

Supplementary_Figures_bbad498

## Data Availability

The data underlying this article are available in the UCSC-TCGA (https://xenabrowser.net/datapages/), CGGA (http://www.cgga.org.cn/download.jsp) and GEO (https://www.ncbi.nlm.nih.gov/geo/) database.

## Author Biographies

**Ning Zhang** is a bioinformatics intern at Shenzhen People’s Hospital, Shenzhen, China. He is also postgraduate students at Shantou University Medical College, China. His research interests include omics data prepeocessing and modeling.

**Fengxia Yang** is a bioinformatics intern at Shenzhen People’s Hospital, Shenzhen, China. She is also postgraduate students at Shantou University Medical College, China. Her research interests include glioma diagnosis and prognosis.

**Pengfei Zhao** is a bioinformatics intern at Shenzhen People’s Hospital, China. He is also a postgraduate student at Southern University of Science and Technology, China. His research focuses on single-cell data analysis and machine learning algorithms.

**Nana Jin**, PhD, is a Postdoctoral Research Fellow at Shenzhen People’s Hospital, China. Her research focuses on NGS data and biological network analysis.

**Haonan Wu** is a Research Assistant at Shenzhen People’s Hospital, Shenzhen, China. His research interests include bioinformatics and omics data analysis.

**Tao Liu** is a Research Engineer at International Digital Economy Academy, Shenzhen, China. His research interests include biostatistics and high-performance computing.

**Qingshan Geng** is a Professor of medicine at Shenzhen People’s Hospital, China. His research interests include big data in healthcare and disease management.

**Xiaojun Yang** is a Professor of medicine at Guangdong Provincial Key Laboratory of Infectious Disease and Molecular Immunopathology, Shantou University Medical College, China. His research interest includes glioma invasion, metastasis and prognosis.

**Lixin Cheng**, PhD, is a Principal Investigator of bioinformatics at Shenzhen People’s Hospital, First Affiliated Hospital of Southern University of Science and Technology, Shenzhen, China. His research interests include modeling and algorithms for big data analysis in medicine and biology.
