# Supplementary material for: MrGPS: an m6A-related gene pair signature to predict the prognosis and immunological impact of glioma patients
Source: Brief Bioinform. 2024 Jan 3;25(1):bbad498. doi: 10.1093/bib/bbad498 (PMC10782913; doi:10.1093/bib/bbad498)
Supplement: Supplementary_Figures_bbad498 [file supplementary_figures_bbad498.docx]

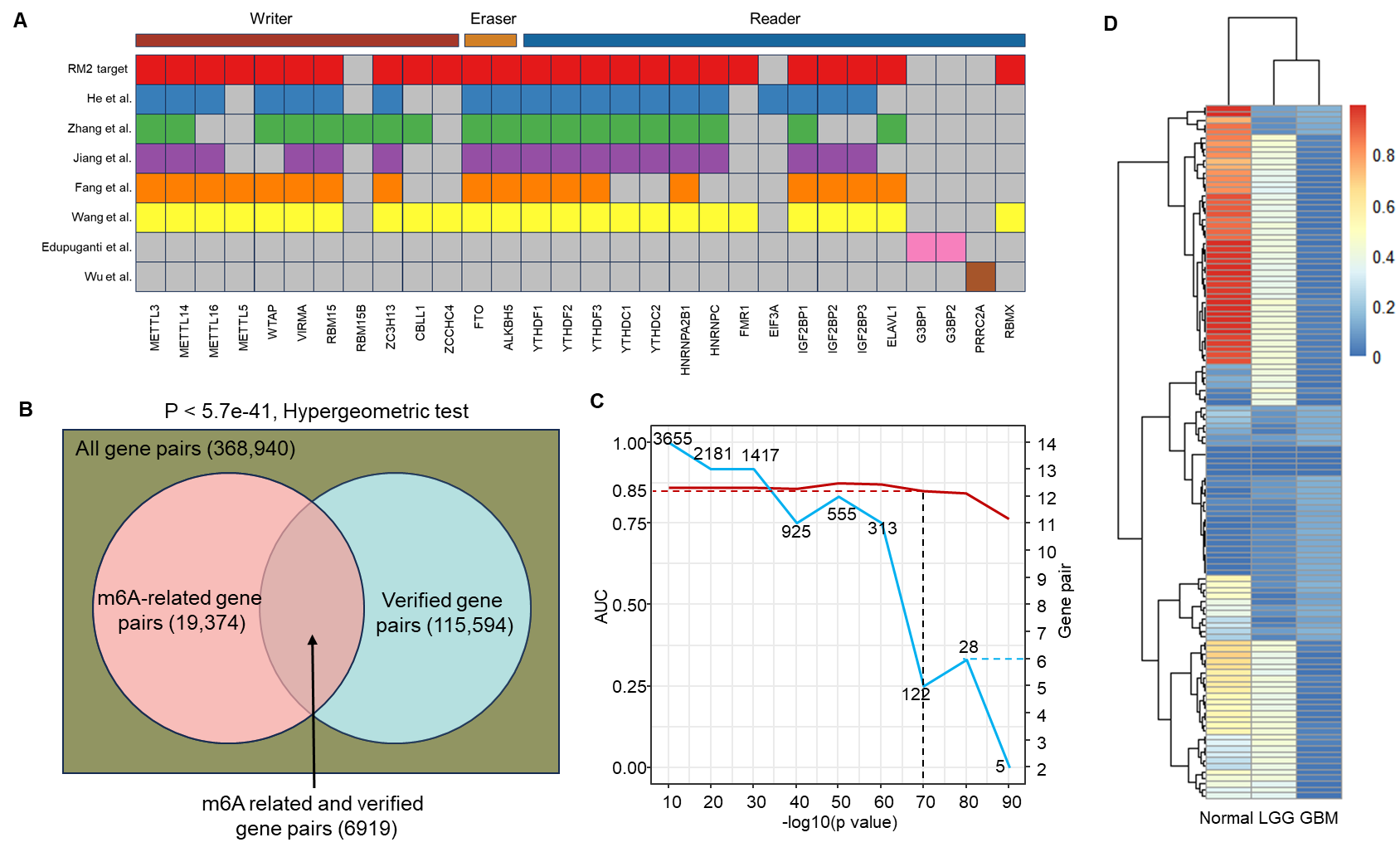


**Figure S1**. The data and parameters used in this study for constructing MrGPS. **(A)** m6A regulators collected from different literatures and databases. **(B)** Hypergeometric test is used to evaluate the significance of the intersection of m6A related gene pairs with experimentally verified gene pairs. **(C)** P-value 1e-70 is selected as the threshold due to it achieves a high AUC (red) using only a few gene pairs (blue). **(D)** Heatmap showing the ratio of each MGPs in which the regulator expressed higher than the target gene in normal brain tissue, LGG and GBM. Color represents the proportion of each m6A related gene pairs in which regulator expressed higher than target gene in the normal brain, LGG and GBM samples, respectively.


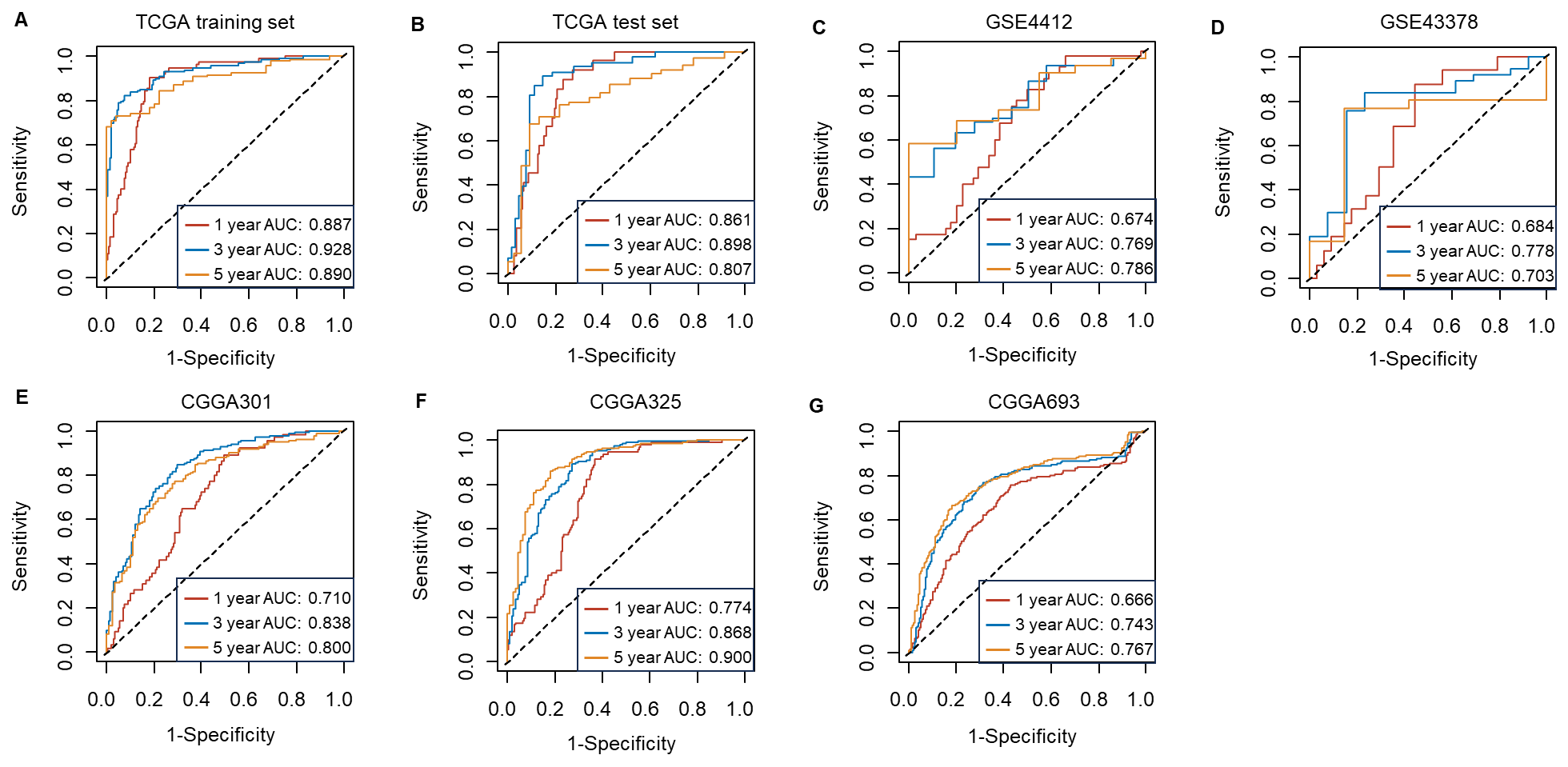


**Figure S2**. Performance validation of the 20-gene prognostic model in seven datasets. Time dependence Receiver-operating characteristic (ROC) curves and AUROC values of 1,3 and 5 year prediction for TCGA training set **(A)**, TCGA test set **(B)**, GSE4412 **(C)**, GSE43378 **(D)**, CGGA301 **(E)**, CGGA325 **(F)** and CGGA693 **(G)**.
